# Drug-Resistant Tuberculosis, KwaZulu-Natal, South Africa, 2001–2007

**DOI:** 10.3201/eid1710.100952

**Published:** 2011-10

**Authors:** Kristina Wallengren, Fabio Scano, Paul Nunn, Bruce Margot, Sandile S.S. Buthelezi, Brian Williams, Alexander Pym, Elisabeth Y. Samuel, Fuad Mirzayev, Wilfred Nkhoma, Lindiwe Mvusi, Yogan Pillay

**Affiliations:** KwaZulu-Natal Research Institute for Tuberculosis and HIV, University of KwaZulu-Natal, Durban, South Africa (K. Wallengren);; World Health Organization, Geneva, Switzerland (F. Scano, P. Nunn, F. Mirzayev);; Department of Health, Pietermaritzburg, South Africa (B. Margot, S.S.S. Buthelezi);; South African Centre for Epidemiological Modelling and Analysis, Stellenbosch, South Africa (B. Williams);; Medical Research Council, Durban (A. Pym);; Inkosi Albert Luthuli Central Hospital, Durban (E.Y. Samuel);; World Health Organization, Harare, Zimbabwe (W. Nkhoma);; Department of Health, Pretoria, South Africa (L. Mvusi, Y. Pillay)

**Keywords:** XDR TB, MDR TB, outbreak, South Africa, incidence, tuberculosis, antimicrobial resistance, drug-resistant, extensively drug-resistant, multidrug-resistant, TB, tuberculosis and other mycobacteria, dispatch

## Abstract

In Africa, incidence and prevalence of drug-resistant tuberculosis have been assumed to be low. However, investigation after a 2005 outbreak of extensively drug-resistant tuberculosis in KwaZulu-Natal Province, South Africa, found that the incidence rate for multidrug-resistant tuberculosis in KwaZulu-Natal was among the highest globally and would be higher if case-finding efforts were intensified.

In Africa, resistance to anti-tuberculosis (TB) drugs has been assumed to be low (*1*). In 2002, the national drug resistance survey showed rates of multidrug-resistant (MDR) TB in South Africa to be 3.0% among all TB cases, 1.6% among new cases, and 6.6% among previously treated cases (*2*). Surveys in other African countries have yielded MDR TB rates of <3% among all TB cases, low compared with >20% reported from former Soviet Union countries; however, MDR TB rates may not be as low as previously estimated (*3**–**7*). In response to a 2005 outbreak of extensively drug-resistant (XDR) TB in KwaZulu-Natal Province, South Africa (*8*), we conducted a retrospective study of the extent and distribution of drug-resistant TB in the province.

## The Study

KwaZulu-Natal Province, population ≈10 million, contains 11 health districts with 68 hospitals in the public health sector. We analyzed existing laboratory records from the only 2 laboratories in the province that conducted culture and drug-sensitivity testing as part of routine clinical care. Since 2001, all samples from patients with MDR TB in the province were tested for susceptibility to second-line anti-TB drugs, except in 2004 and 2005, when 82% (1,143) and 55% (1,277) of samples, respectively, were not tested for fluoroquinolones. From 2006 on, all culture-positive cases in the province were tested for susceptibility to first-line and second-line anti-TB drugs. We reviewed laboratory results from 2001 through 2007 and determined the number of MDR TB and XDR TB cases for each district. To provide context for the 2005 XDR TB outbreak at the Church of Scotland Hospital (COSH) in KwaZulu-Natal Province (*8*), we also analyzed geographic distribution and time trends. Per definition, the number of MDR TB cases includes all XDR TB cases.

According to national guidelines, samples for culture should be collected from persons who are being initially examined for retreatment, those for whom treatment has failed, those whose sputum smear results are negative but who have clinical signs of TB and do not respond to antibacterial drug treatment (excluding TB treatment), and those suspected of having HIV and TB co-infection. Despite the guidelines, the intensity with which cultures were requested varied among districts. We compared culture-taking practices with prevalence of MDR TB per district during the same time frame. Culture-taking practices were derived from the number of patients for whom a culture was requested during 12 months after March 2006 and were analyzed per district and per 100,000 population. The proportion of identified MDR TB patients who received treatment with second-line anti-TB drugs was calculated by dividing the number of patients with laboratory-confirmed MDR TB by the number of patients admitted to King George V Hospital, the only TB hospital in the province that treated patients with MDR TB during the same period. (Detailed methods available from K.W. upon request.)

In 2007, a total of 2,799 cases (28 cases/100,000 population) of MDR TB were identified in KwaZulu-Natal. TB prevalence was 1,200 cases/100,000 population, and MDR accounted for 2.3% of reported cases in the province (http://arxiv.org/abs/1107.1800). XDR TB cases accounted for 9.6% of MDR TB cases (Table).

**Table:** Cases of MDR and XDR TB, KwaZulu-Natal Province, South Africa, 2007*

District	All TB cases	MDR TB cases	XDR TB cases	MDR/all TB cases, %	XDR/MDR cases, % (95% CI)	No. MDR cases/ 100,000 population
eThekwini	45,019	1,014	64	2.3	6.3 (4.9–8.0)	31.7
Ugu	10,618	226	9	2.1	4.0 (1.8–7.4)	31.0
uMgungundlovu	10,687	247	36	2.3	14.6 (10.4–19.6)	25.7
uThukela	6,129	69	8	1.1	11.6 (5.1–21.6)	10.1
Umzinyathi	5,522	226	120	4.1	53.1 (46.4–59.7)	47.8
Amajuba	3,578	61	2	1.7	3.3 (0.4–11.3)	12.6
Zululand	8,478	171	6	2.0	3.5 (1.3–7.5)	20.5
Umkhanyakude	6,991	337	4	4.8	1.2 (0.3–3.0)	56.8
Uthungulu	11,876	233	11	2.0	4.7 (2.4–8.3)	25.4
iLembe	5,007	118	6	2.4	5.1 (1.9–10.7)	20.3
Sisonke	5,313	61	4	1.1	6.6 (1.8–15.9)	12.9
Total	119,218	2,799	270	2.3	9.6 (8.6–10.8)	28.2

*MDR, multidrug-resistant; XDR, extensively drug-resistant; TB, tuberculosis; CI, confidence interval.

In 2007, MDR TB in the districts ranged from 10 (uThukela) to 57 (Umkhanyakude) cases per 100,000 population (Table). Incidence of MDR TB was highest for Umkhanyakude and Umzinyathi districts (location of COSH) (*8*). The proportion of MDR TB cases that were XDR TB cases also varied among districts (1.2%–53.1%). The 2 districts with the highest level of MDR TB (Umkhanyakude and Umzinyathi) had the highest and the lowest XDR TB prevalence, respectively (Table).

**Figure 1:**
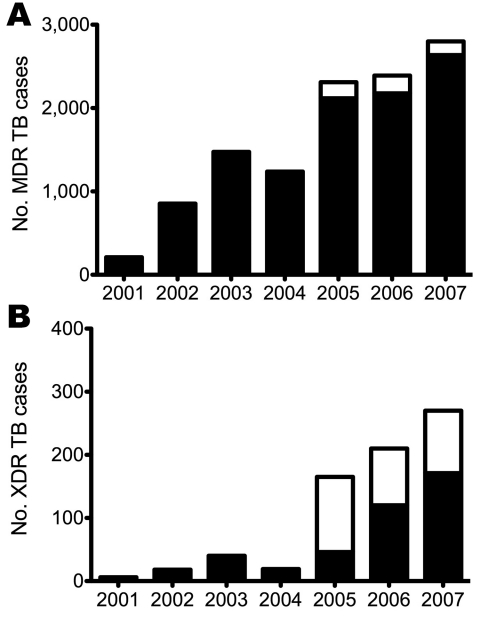
Prevalence of drug-resistant tuberculosis (TB) in the 11 districts of KwaZulu-Natal, South Africa, 2001–2007. A) Multidrug-resistant (MDR) TB; B) extensively drug-resistant (XDR) TB. White bar sections, Church of Scotland Hospital; black bar sections, the rest of KwaZulu-Natal Province.

**Figure 2:**
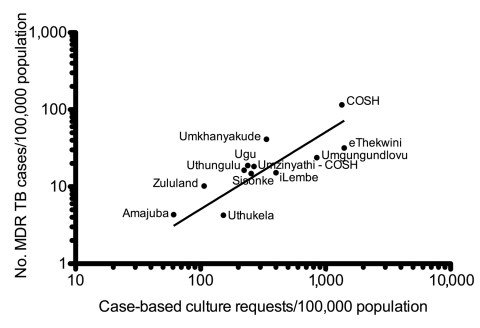
Culture-taking practice correlation with identified multidrug-resistant tuberculosis (MDR TB) prevalence in the 11 districts of KwaZulu-Natal Province and in the Church of Scotland Hospital (COSH), South Africa, 2001–2007. Because of the high level of culture-taking at COSH, COSH data were subtracted from the Umzinyathi district data. Black line indicates the level of MDR TB that would be identified if the whole province requested the same number of culture and sensitivity testing as COSH.

From 2001 to 2007, the level of MDR TB increased >10-fold, from 216 cases to 2,799 cases, respectively (Figure 1, panel A), and XDR TB increased from 6 cases to 270 cases, respectively (Figure 1, panel B). In part, the increase reflects increased sampling, which tripled between 2002 and 2007, from ≈40,000 to >120,000 samples tested each year. The variation in MDR TB prevalence between districts could be attributed to differences in culture-taking practice (Spearman correlation coefficient 0.82; p = 0.001) (Figure 2). XDR TB prevalence, expressed as proportion of MDR TB, was not affected by culture-taking practices because all positive cultures were tested for first-line and second-line anti-TB drugs.

XDR TB has been in KwaZulu-Natal Province since 2001 and was first identified in eThekwini. XDR TB increased rapidly in 2005 when the outbreak was identified, and 72% of all XDR TB cases in the province were at COSH (Figure 1, panel B). As a result of this outbreak, culture-taking practices in the rest of the province increased, and in 2007, the proportion of identified XDR TB cases in the province that were at COSH had decreased to 37%. Excluding Umzinyathi, an average of 6.5% of all MDR TB cases were also XDR TB.

Within 12 months after March 2006, only 32% (896) of 2,784 patients with laboratory-identified MDR TB had received treatment with second-line drugs. During 2005–2006, the average time between sputum collection and admission to King George V Hospital was 16 weeks. The delay reflects turnaround time for culture and sensitivity testing (4–6 weeks), delay in returning results to the referring health facility (not all sites have Internet access), tracing of patients, and hospital admission waiting time.

## Conclusions

In 2007, South Africa ranked fourth among countries with the highest estimated number of MDR TB cases (*9*). Within South Africa, KwaZulu-Natal Province had the highest prevalence of drug-resistant TB and accounted for 38% (2,799) of 7,350 MDR TB cases and 50% (270) of 536 XDR TB cases in the country (*10**,**11*).

The reported MDR TB incidence rate per 100,000 population of KwaZulu-Natal is among the highest worldwide. Districts Umkhanyakude and Umzinyathi reported 57 and 48 cases/100,000 population, respectively, more than the highest previously reported estimate of 35 cases/100,000 population in Karakalpakstan, Uzbekistan (*12*). Because many patients never have a sample taken for culture and sensitivity testing, the identified level of MDR TB is an underestimate.

After the 2005 outbreak, COSH increased vigilance for drug resistance, and culture and sensitivity testing was conducted for all patients with suspected TB. If other clinics and hospitals in the province requested as many cultures as COSH, MDR TB in KwaZulu-Natal would amount to 68 cases/100,000 population, with an estimated 6,750 MDR TB cases and 526 XDR TB cases per year, 2–3× more than currently identified (Figure 2). Furthermore, the increase in XDR TB during 2006–2007 suggests ongoing transmission of XDR TB.

Study limitations include unavailability of data to assess trends in culture-taking practices, so we could not evaluate how culture-taking practices may have influenced the increase in reporting of MDR TB over time. Also unavailable were data for categorizing MDR TB cases as new or retreatment.

Our findings show that incidence rates of MDR TB in KwaZulu-Natal could be higher than previously estimated. Since 2007, steps have been taken to validate and implement rapid diagnostic tests for all TB patients in the province and to increase access to MDR TB treatment by increasing bed capacity and decentralizing the MDR TB treatment program. The challenge is ensuring that all patients with a new diagnosis of MDR TB have access to treatment. Similarly, ongoing transmission of TB must be reduced by implementation of sound infection control measures.
